# Eyelid Amyloidosis: A Rare Condition With Diagnostic Challenges

**DOI:** 10.7759/cureus.90107

**Published:** 2025-08-14

**Authors:** Omar Alobaidan, Naif Al Sulaiman, Abeer Alharbi, Nouf Almushayt

**Affiliations:** 1 Oculoplastics, King Khaled Eye Specialist Hospital, Riyadh, SAU; 2 Ophthalmology, Ohud Hospital, Medina, SAU; 3 Ophthalmology, Abha Maternity and Children Hospital, Abha, SAU

**Keywords:** dystopia, lid swelling, meibomian gland atrophy, ptosis, systemic amyloidosis

## Abstract

We present a rare and challenging case of a man who presented with mild mechanical ptosis and inferior dystopia, diagnosed with eyelid amyloidosis. A 40-year-old healthy man presented with a painless mass on his left upper eyelid that started one year ago. He noted that the mass increases in size when he eats meat, but there has been no overall progression over time. He experienced inferior dystopia with left upper eyelid fullness, swelling, and mild mechanical ptosis of the left eye. The measurements were as follows: marginal reflex distance 1 was 5 mm (oculus dexter, OD) and 4 mm (oculus sinister, OS); palpebral fissure height was 13 mm (OD) and 10 mm (OS); Hertel exophthalmometer reading at 105 was 22 mm (OD) and 23 mm (OS). CT was done with the impression of a dermoid cyst. It showed complex mass lesions of mixed fat, soft tissue, and calcification, predominantly involving the left upper eyelid with deeper extension into the superior compartment of the extraconal part of the fat of the orbit, close to the superior oblique levator muscle, with subsequent mild enlargement of the extraconal muscle group bilaterally, predominantly seen on the left side. The excisional biopsy showed amyloidosis. Amyloidosis is uncommon, progressive, and slowly associated with significant ocular morbidity. The diagnosis might be delayed because of an unusual presentation.

## Introduction

Studying localized eyelid amyloidosis is essential for accurate diagnosis and appropriate management, given the condition’s rarity, potential for misdiagnosis, and varied clinical presentations [1-3]. Systemic amyloidosis is a rare disorder characterized by the abnormal accumulation of misfolded proteins, known as amyloid fibrils, in various organs and tissues throughout the body. These amyloid fibrils are insoluble and can disrupt normal organ function, leading to organ damage and dysfunction.

There are several types of systemic amyloidosis, including amyloid formed from immunoglobulin light chain (AL) and amyloid transthyretin (ATTR) amyloidosis. AL amyloidosis is caused by the abnormal production and deposition of immunoglobulin light chains, which are components of antibodies, whereas ATTR amyloidosis is caused by the deposition of abnormal transthyretin proteins.

Systemic amyloidosis can affect multiple organs, including the liver, kidneys, heart, nervous system, and gastrointestinal tract. The symptoms and severity of the disease can vary depending on the organs affected and the extent of amyloid deposition.

Diagnosis of systemic amyloidosis often involves a combination of clinical evaluation, laboratory tests, imaging studies, and tissue biopsy. Localized amyloidosis of the eyelid is encountered infrequently in clinical practice, but its recognition is crucial because of its potential to mimic other common eyelid conditions, leading to misdiagnosis and delayed treatment [4,5].

High clinical suspicion, accurate diagnosis through tissue biopsy, and awareness of its diverse presentations are essential for prompt management and to exclude systemic involvement and ensure appropriate patient care, because the disease can be progressive and potentially life-threatening if left untreated [4,5].

The clinical manifestations of primary localized amyloidosis typically include a tarsal mass with atypical tarsal plate thickening, which is a rare feature often resembling a bone tumor due to extensive calcifications. Notably, amyloidosis can lead to meibomian gland atrophy and may be associated with evaporative dry eye disease. Diagnosis involves evaluating tarsal thickening with histology and assessing for amyloid deposition using Congo red stain and metachromasia with methyl violet stain. This localized amyloidosis in the absence of systemic involvement suggests a local immunocyte disorder as the underlying cause [2]. The diagnosis is usually confirmed through a biopsy [6,7]. Localized amyloidosis masses in the lids are considered rare entities, often presenting with unique clinical manifestations such as proptosis, ptosis, recurrent subcutaneous hemorrhages, and dry eyes. The condition’s rarity is highlighted by the low reported frequency of such occurrences in the medical literature, underscoring the need for further research and documentation [3,8]. In the literature, localized amyloidosis involving the eyelids is considered rare, with only 4% of such cases encountered in the orbital region. Additionally, only 1.3% of cases involve deposition in the extraocular muscles [8]. The rarity of this condition highlights the importance of further research and documentation to enhance understanding, recognize patterns, facilitate prompt diagnosis, and determine optimal management strategies. By delving deeper into the clinical presentations, diagnostic challenges, and treatment outcomes, researchers can contribute valuable insights that may improve patient care and outcomes in such uncommon cases [8]. Thus, we present this rare and challenging case of a man who came in with mild mechanical ptosis and inferior dystopia and was diagnosed with eyelid amyloidosis.

## Case presentation

**Figure 1:**
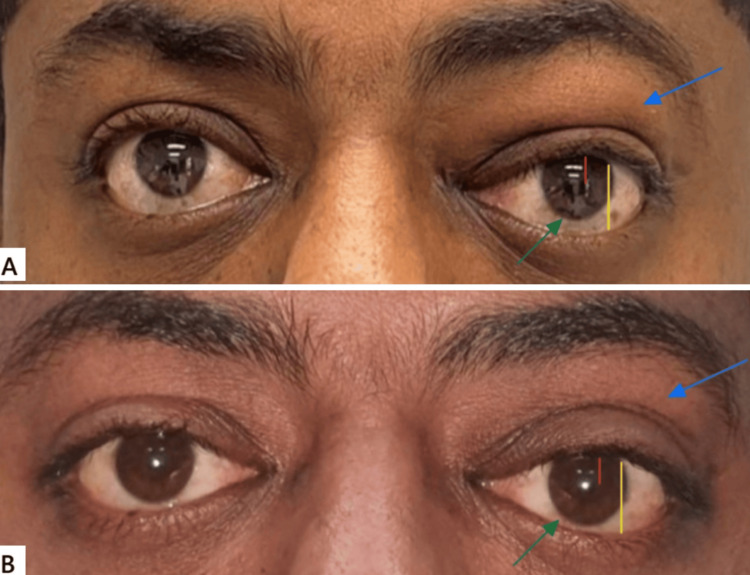
(A) Preoperative image showing inferior dystopia with left upper lid fullness (blue arrow), mild mechanical ptosis in the left eye (yellow and red lines), and sclera showing inferior (green arrow). (B) The postoperative image illustrating a marked decrease in the fullness of the left upper lid (blue arrow), no mechanical ptosis (yellow and red lines), and no scleral show (green arrow)

A 40-year-old medically free man came with a painless mass in the left upper lid that started one year previously; the man mentioned that it increased in size when he ate meat but did not progress over time (Figure 1A). The mass was soft, central, and mobile. It caused him inferior dystopia with left upper lid fullness (Figure 1A, blue arrow), swelling, mild mechanical ptosis in the left eye, and no increase of the anterior orbital mass with the Valsalva maneuver.

**Figure 2:**
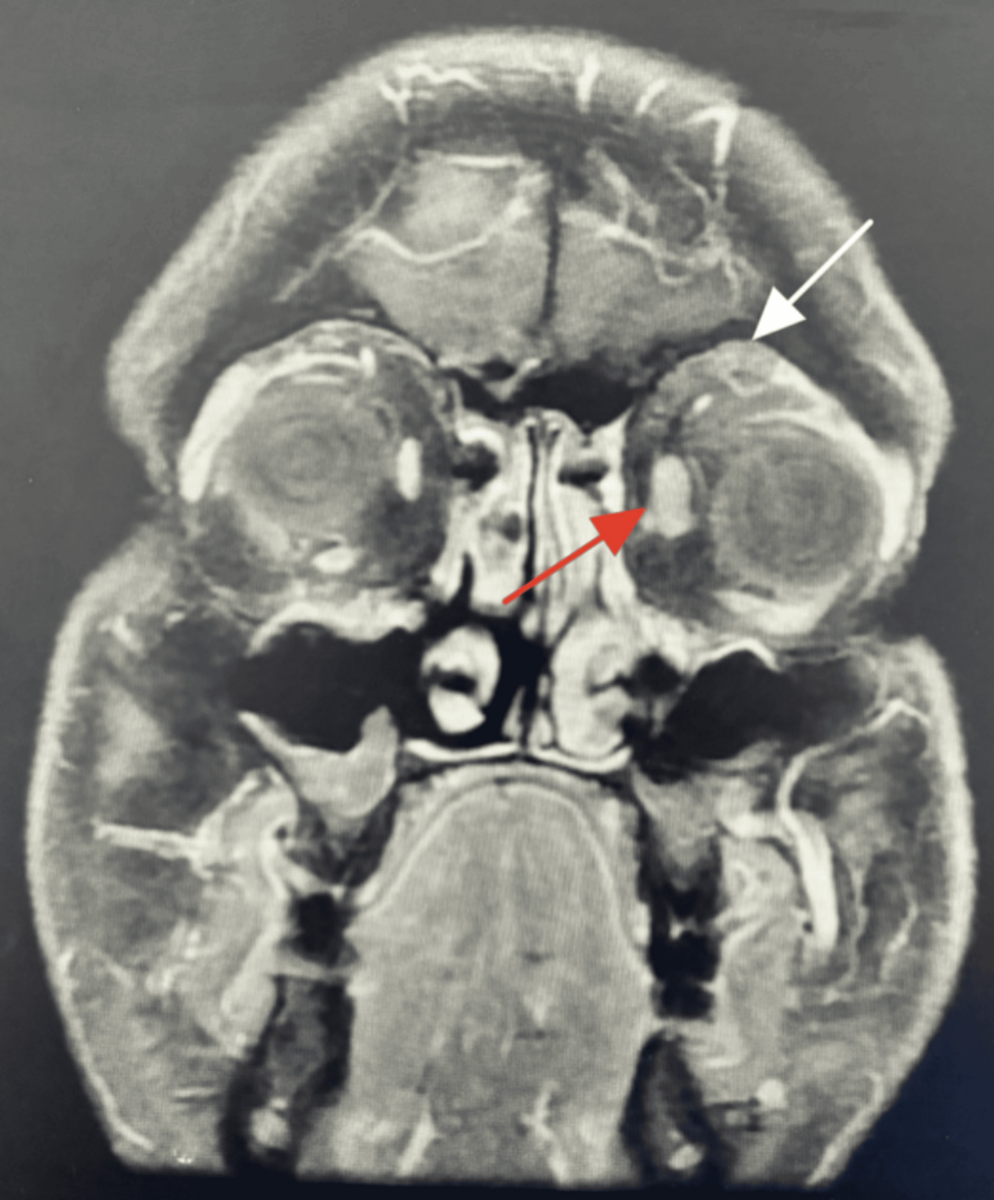
T1 coronal plane showing a mass lesion, with mixed signal intensity intermediate to low on T1-weighted image in the superior part of the left orbit and preseptal in location (white arrow). The left medial rectus muscle is mildly enlarged (red arrow)

The margin to reflex distance 1 was 5 mm for the right eye (oculus dexter, OD) and 4 mm for the left eye (oculus sinister, OS; Figures 1A, 1B, red line), whereas the palpebral fissure height was 13 mm (OD) and 10 mm (OS; Figures 1A, 1B, yellow line). The Hertel exophthalmometer at 105 was 22 mm (OD) and 23 mm (OS). An MRI with contrast was done, and the pattern of enhancement of the mass lesion did not support the pattern of enhancement of a neurogenic tumor, nor the appearance of a plexiform neurofibroma. The observed features are thought to be associated with chronic inflammatory changes and amyloidosis, with involvement of the related part of the supraorbital nerve (see Figure 2, white arrow). In the T1 coronal plane, a mass lesion is present in the superior part of the left orbit, exhibiting mixed signal intensity that ranges from intermediate to low on the T1-weighted image and is located in the preseptal area (see Figure 2, red arrow). Additionally, the left medial rectus muscle appears to be mildly enlarged.

The patient was then booked for excisional biopsy through the lid crease, and the result of the biopsy showed amyloidosis.

## Discussion

Amyloidosis is an uncommon occurrence, with its worldwide incidence estimated to be five to nine cases per million patients yearly [9]. Amyloidosis is characterized by the accumulation of insoluble amyloid fibrils (an abnormal protein) in body tissues, causing functional impairment. Its presentation can either be localized to a single site, which is infrequent in the periocular area, or it can be systemic, in which case it accumulates throughout the body (commonly in the heart and kidneys) [9]. Periocular involvement has been unusually reported in the literature. The amyloid deposition often occurs in the lacrimal gland, orbital soft tissues, extraocular muscles, eyelid skin, conjunctiva, or cornea, but might also be seen in the anterior chamber, iris, vitreous, or optic nerve [9]. Periocular involvement is noted in only 4% of the cases, with 1.3% involving deposition in extraocular muscles [8].

The diverse clinical presentations of periorbital and ocular amyloidosis might cause a delay in diagnosis. Eyelid amyloidosis is a rare presentation, and it might present differently in each patient [10]. Our patient presented with upper lid fullness with a soft, central, mobile mass, causing inferior dystopia with mild ptosis. In one case report, the patient presented with a lid mass and trichiasis after excision, along with typical amyloid deposits without systemic association [8]. Another case report found that two patients presented with irregular tarsal thickening and severe ptosis, and after incision, a biopsy showed amyloid deposits with meibomian gland atrophy [11]. In a third case report, an older patient presented with a nodular ulcerative lesion affecting the lid margins and the medial canthal area, involving the conjunctiva and caruncle without systemic associations [2]. All these previous cases presented with different types of lid involvement.

In these patients, ptosis could be explained either by mechanical ptosis of the lid due to the weight of amyloid itself or infiltration and stretching of extraocular muscles such as Muller’s muscle and the levator, as has been mentioned in previous studies [2]. Amyloidosis can cause muscle necrosis in the levator muscles and compressive optic neuropathy, and lacrimal gland involvement can cause keratoconjunctivitis sicca, whereas meibomian gland atrophy can cause dryness. This leads to either muscle necrosis or mechanical dysfunction [2]. Other features such as lacrimal gland enlargement, keratoconjunctivitis sicca, eyelid skin papules, and compressive optic neuropathy were absent in our patient.

Diagnosis of orbital and periocular amyloidosis is a challenge because of its different clinical presentations, and a biopsy is needed to reveal apple‐green birefringence on Congo red stain. [8] In the case of localized orbital amyloidosis, systemic involvement must be ruled out [9].

Amyloidosis occurs as a result of the slow progression of accumulated material, so the management options are various and must be individualized. Depending on the site involved, conservative management might be an option. Advanced cases, especially in the periocular area, are better managed by surgical debulking, as in our patient with mechanical ptosis [11]. Besides controlling the underlying disease, if any, the main aims of surgical treatment are to maintain function and cosmetic appearance [12].

## Conclusions

Amyloidosis is uncommon and progressing slowly when associated with significant ocular morbidity. The diagnosis might be delayed because of unusual presentation. Lid involvement can present in different ways. We reported in this case a different orbital presentation of amyloidosis.
